# The complete chloroplast genome sequence of *Limonium sinense* (Plumbaginaceae)

**DOI:** 10.1080/23802359.2019.1710286

**Published:** 2020-01-14

**Authors:** Jianfang Li, Bei Xu, Qian Yang, Ting Wang, Qianyang Zhu, Yini Lin, Zhan-Lin Liu

**Affiliations:** Key Laboratory of Resource Biology and Biotechnology in Western China (Ministry of Education), College of Life Sciences, Northwest University, Xi’an, Shaanxi, China

**Keywords:** *Limonium sinense*, plastome, phylogeny, Plumbaginaceae

## Abstract

*Limonium* is a species-rich genus in Plumbaginaceae. In this study, we determined the complete chloroplast genome of *Limonium sinense* for potential phylogenomic analysis in the future. The plastome is 174,033 bp in length, with a large single-copy region (LSC) of 96,128 bp, a small single-copy region (SSC) of 13,517 bp and a pair of inverted repeat regions (IRs) of 32,194 bp. It contains 132 genes, including 84 protein-coding genes, 37 tRNA genes, 8 rRNA genes, and 3 pseudogenes. The overall GC content is 36.7%, while the corresponding values for the LSC, SSC, and IR region are 34.9, 31.1, and 40.6%, respectively. The phylogenetic analysis supported that *L. sinense* was clustered together with *Plumbago auriculata* in family Plumbaginaceae.

*Limonium* is the most species-rich genus of Plumbaginaceae family with about 300–600 species, many of which are endemic with a very restricted range. The infrageneric relationships have been revised several times and are not completely identified due to similar morphological characters and limited taxon sampling (Koutroumpa et al. 2018). Recently, analysis of chloroplast genome data provides sufficient phylogenetic signals for the rapidly divergent groups with a tremendous amount of members (Guo et al. 2017). In the present study, we determined the complete chloroplast genome of *Limonium sinense* (Girard) Kuntze, a perennial herb distributed in the coastal provinces of China as well as in Vietnam. This species is a popular garden flower in China and is also used as a traditional Chinese medicine with antitumor and antiviral activities (Kuo et al. 2002; Tang et al. 2012). We expect that the plastome will benefit the phylogenetic works of Plumbaginaceae in the future.

Individuals of *L. sinense* were collected from Ningxia of China (N38.58°, E105.96°). The voucher (2017071) was deposited at the Evolutionary Botany Laboratory (EBL), Northwest University, China. Genomic DNA was isolated from one individual and sequenced with the high-throughput Illumina sequencing platform. The assembly and annotation of the chloroplast genome followed the method published previously (Li et al. 2019; Peng et al. 2019) with the reference of *Plumbago auriculata* (NC041245). The plastome sequence has been deposited into GenBank with the accession number MN599096.

The complete chloroplast genome of *L. sinense* is 174,033 bp in length, with a large single-copy region (LSC) of 96,128 bp and a small single-copy region (SSC) of 13,517 bp, separated by a pair of inverted repeat regions (IRs) of 32,194 bp. It contains 132 genes, including 84 protein-coding genes, 37 tRNA genes, 8 rRNA genes, and 3 pseudogenes. Twenty-nine protein-coding genes, 17 tRNA genes, and 4 rRNA genes are transcribed in a clockwise direction, while others are transcribed counterclockwise. Seventeen genes are duplicated in the IRs. Among the annotated genes, 14 contain a single intron and three (*ycf3*, *rps12*, and *clpP*) harbor two introns. The *rps12* gene was predicted to be trans-spliced, with the 5′ end located in the LSC region and the duplicated 3′ end in the IR region. The overall GC content of *L. sinense* plastome is 36.7%, and the GC content of the IR regions (40.6%) is higher than those of the LSC and SSC regions (34.9 and 31.1%, respectively).

For lacking information on Plumbaginaceae plastomes, the phylogenetic position of *L. sinense* was just analyzed at the order level. All protein-coding genes of 19 representative plastomes from Caryophyllales were used to construct the maximum-likelihood tree with two taxa in Santalales as outgroup (Peng et al. 2019). The phylogenetic analysis indicated that *L. sinense* was clustered together with *P. auriculata* in the family of Plumbaginaceae (Figure 1) and the interfamilial topologies were consistent with the tree constructed with chloroplast DNA fragments proposed by Schäferhoff et al. (2010).

**Figure 1.**
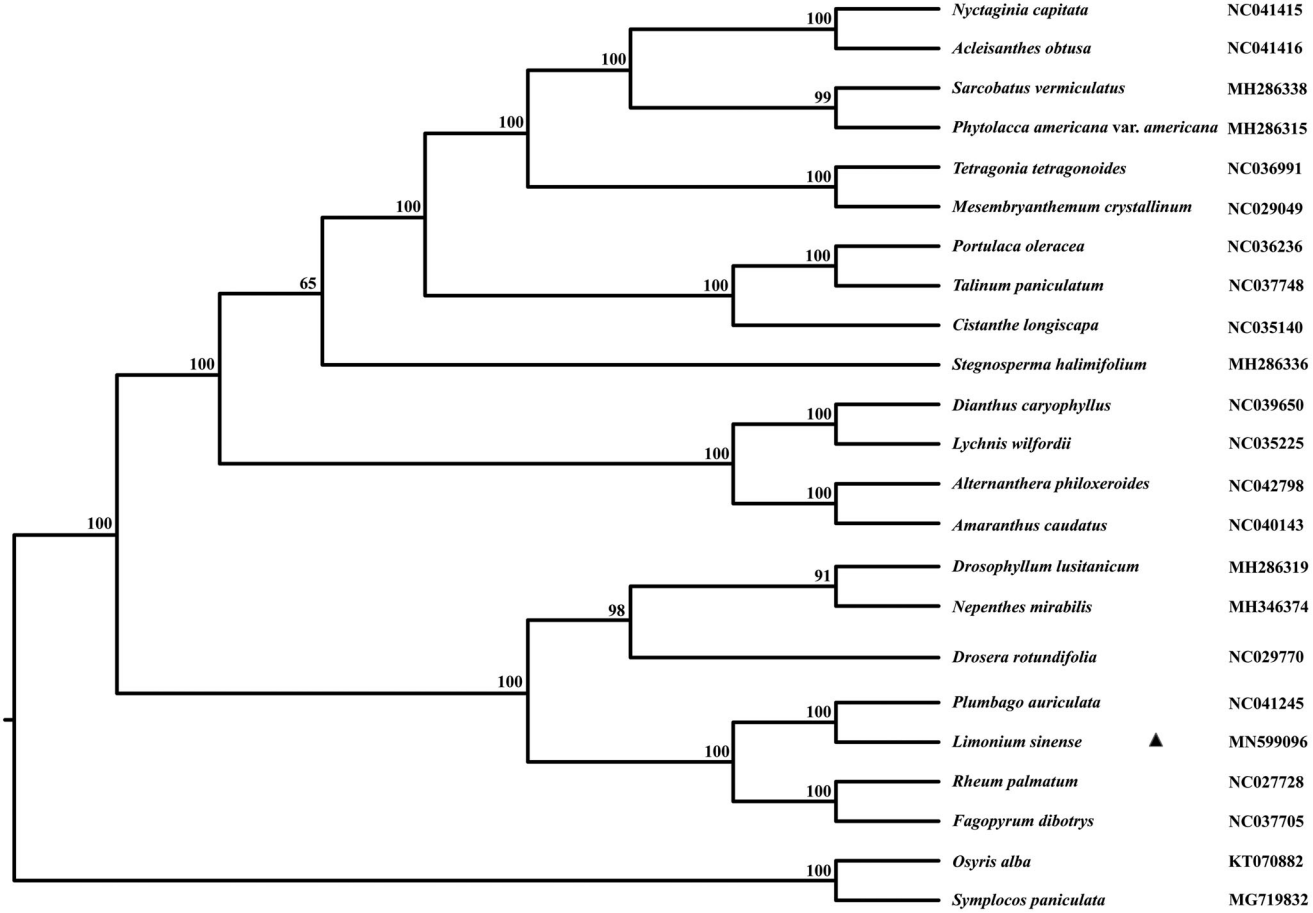
The maximum-likelihood tree of 20 representatives in Caryophyllales constructed with the protein-coding genes. The bootstrap values were based on 1000 replicates.
